# First-Principles Study of Hazardous Gas Molecule Adsorption on Janus MoSTe Monolayer Modified with Surface Vacancy Defect

**DOI:** 10.3390/nano16100621

**Published:** 2026-05-18

**Authors:** Yuhui Zhu, Sheng Xu, Qiang Wang, Yanni Gu, Xiaoli Zhang, Xiaoshan Wu

**Affiliations:** 1 School of Metallurgy Engineering, Jiangsu University of Science and Technology, Zhangjiagang 215600, China; zyh689554@163.com (Y.Z.);; 2 National Laboratory of Solid State Microstructures and School of Physics, Nanjing University, Nanjing 210093, China

**Keywords:** MoSTe monolayer, density functional theory, vacancy defect, gas sensing, electronic properties

## Abstract

Novel highly sensitive two-dimensional gas-sensing materials for detecting hazardous gases are crucial for human health, climate protection, and industrial development. In this study, density functional theory (DFT) was employed to investigate the adsorption and sensing properties of four representative hazardous gas molecules (NO, NO_2_, F_2_, and Cl_2_) on pristine and vacancy-defective (S vacancy and Te vacancy) Janus MoSTe monolayer. The introduction of a vacancy into the MoSTe monolayer significantly reduces the adsorption distances and enhances the adsorption energies and charge transfers. Notably, an S vacancy induces a transition in the adsorption behaviors of NO, NO_2_, and Cl_2_ on MoSTe from physisorption to chemisorption, and a Te vacancy leads to strong physisorption of NO and NO_2_ on the MoSTe monolayer. Electronic structure analysis further reveals that gas molecule adsorption can modulate band gaps. Adsorption of F_2_ and Cl_2_ on the Te surface of pristine MoSTe converts the indirect bandgap into a direct bandgap. However, the calculation results for O_2_ adsorption indicate that the S and Te vacancies in Janus MoSTe may be readily occupied by O_2_, suggesting that it is not a good sensing material under atmospheric conditions. This study provides valuable theoretical insights and guidance for future experiments on vacancy-defective Janus MoSTe monolayer.

## 1. Introduction

Rapid industrialization of society has resulted in a growing volume of hazardous gases being emitted into the atmosphere, representing a major risk to the environment and human health. For example, industrial production and fossil fuel combustion release substantial quantities of hazardous nitrogen oxides (NO and NO_2_) [1]. These nitrogen oxides contribute to various environmental problems, including acid rain, haze, and photochemical smog [2]. Additionally, they contribute to an increased morbidity of respiratory diseases, thereby negatively impacting human health [3]. Occupational exposure to toxic halogen gases may occur during industrial production and maintenance activities [4]. As the most reactive nonmetals, halogens present a serious threat to human health [5]. For instance, inhaling low concentrations of F_2_ can cause ligament calcifications and osteosclerosis, while higher concentrations may prove fatal [6]. Exposure to low concentrations of Cl_2_ can irritate the eyes, skin, and respiratory tract [7]; inhaling higher concentrations may result in toxic pneumonitis, pulmonary edema, and even death [8]. Therefore, the development of materials for highly sensitive detection of these hazardous gases is critical for human health, the global climate, and industrial development.

Since the discovery of graphene [9,10], extensive research has been conducted on two-dimensional (2D) materials in gas sensing [11,12]. Transition metal dichalcogenides (TMDs), as typical 2D materials, have gained considerable attention in recent research on novel hazardous gas-sensing materials, owing to their significant advantages, including a large surface-to-volume ratio, high surface activity, and tunable electronic properties [13,14,15]. Pham et al. demonstrated that single-layer MoS_2_ can be used for highly sensitive NO_2_ gas sensing by inducing a photocurrent [16]. Wu et al. found that, under ultraviolet illumination, a MoTe_2_ gas sensor achieves ultrasensitivity and full reversibility [17]. Xu et al. successfully synthesized WS_2_ nanosheets, which exhibit outstanding sensing performance toward NO_2_ [18]. Guo et al. indicated that WSe_2_ nanosheets show promise as ideal candidate materials for ultrasensitive NO_2_ sensing applications [19].

Generally, when studying the adsorption and sensing properties of TMDs toward hazardous gases, pristine TMDs often exhibit weak interactions with these hazardous gases [20,21]. This may limit the sensitivity and selectivity of hazardous gas sensing. However, the interaction between TMDs and hazardous gases can be effectively enhanced by introducing defects [22], doping with metals [23], applying strain [24], or applying an external electric field [25]. But it is worth noting that stronger interactions do not necessarily benefit sensing. According to transition state theory [26], the greater the absolute value of the adsorption energy, the longer the recovery time. Cui et al. reported [27] that when the adsorption energy ranges from −1.6 eV to −0.8 eV, the adsorption system exhibits excellent adsorption and desorption behavior, making it suitable for highly sensitive, low-cost chemical resistance-type gas sensors with good recovery performance. When the adsorption energy is greater than −0.8 eV, physical adsorption dominates, enabling the development of field-effect transistor gas sensors, which are characterized by high accuracy and rapid response but at a high cost. When the adsorption energy is less than −1.6 eV, the recovery time becomes extremely long, thereby limiting its application in sensing.

Over the past few years, Janus TMDs, a novel class of TMDs, have been successfully synthesized using methods such as chemical vapor deposition (CVD) and pulsed laser deposition (PLD) [28,29,30,31]. Compared to conventional TMDs, Janus TMDs exhibit unique physicochemical properties arising from broken structural symmetry and built-in dipole moments and electric fields [32]. As shown in Figure S1, taking Janus MoSTe as an example, compared to conventional TMDs such as MoS_2_ and MoTe_2_, the differences in electronegativity and covalent radii between S and Te atoms lead to a redistribution of valence charge. Specifically, the S atom exhibits a Bader charge of 6.58 e, indicating charge accumulation, whereas the Te atom shows a Bader charge of 6.21 e, corresponding to charge depletion. This asymmetric charge distribution, together with the intra-layer charge transfer between the S and Te surfaces, generates a built-in dipole moment and electric field in Janus MoSTe (Figure S2). The dipole moment and electric field may influence the interactions between polar gas molecules and the surface during adsorption, thereby modulating their adsorption strength on the MoSTe surface. Furthermore, vacancy defects are inevitable in Janus TMDs and typically act as more reactive adsorption sites during the synthesis process [20,33]. Consequently, the effect of vacancy defects on the adsorption and sensing properties of hazardous gases in Janus TMD monolayers has garnered increasing research attention. Dou et al. achieved high sensitivity for NO, CO, and O_2_ adsorption on Janus WSTe by introducing vacancy defects [34]. Paul et al. predicted that WSeTe with a Se atom vacancy is an ideal candidate material for electrochemical gas sensors [20]. Chaurasiya and Dixit also investigated the superior adsorption performance of Janus MoSSe, featuring S-Se vacancies or an S vacancy defect, toward NH_3_, NO_2_, and NO [35]. As a Janus TMDs with the same structure as gas-sensing materials such as WSTe, WSeTe, and MoSSe, the MoSTe monolayer with a vacancy may be a potential candidate material for detecting hazardous gases. However, recent studies on Janus MoSTe in gas sensing have mainly concentrated on its adsorption behavior toward organic compounds [36,37], electrical equipment characteristic gases [38,39,40], and industrial exhaust gases [41]. There remains a lack of studies on the impact of vacancy defects in Janus MoSTe on the adsorption and sensing properties of common hazardous gas molecules. Therefore, it is highly meaningful to investigate the influence of vacancy defects on the adsorption and sensing properties of common hazardous gases on Janus MoSTe.

In this paper, we investigate the adsorption and sensing properties of Janus MoSTe toward common hazardous gases, including NO, NO_2_, F_2_, and Cl_2_. Selecting these two distinct gas categories, nitrogen oxides (NO and NO_2_) and halogen gases (F_2_ and Cl_2_), this study can comprehensively evaluate the differences in adsorption performance between these two gas categories in Janus MoSTe, and the varying effects that the adsorption of the two gas categories has on electronic properties. By calculating adsorption distances, adsorption energies, charge transfers, work functions, and recovery times, we examined the adsorption properties of hazardous gas molecules on pristine and vacancy-defective Janus MoSTe monolayer. Additionally, we conducted a detailed analysis of changes in electronic properties before and after adsorption. We also discuss the practical limitations imposed by the O_2_ adsorption on the vacancy in sensing applications under atmospheric conditions. This study investigates the role of vacancy defects in modulating the material’s adsorption properties and the impact of hazardous gas adsorption on its electronic properties. Importantly, it also elucidates the limitations that may be faced under real sensing conditions. Our research provides valuable theoretical insights and guidance for future experiments on vacancy-defective Janus MoSTe.

## 2. Computational Details and Methods

All first-principles calculations in this study were conducted using the Vienna Ab initio Simulation Package (VASP) [42], based on Density Functional Theory (DFT). The electron-ion interactions were described by the Projector-Augmented Wave (PAW) potential [43], while the exchange-correlation interactions were treated with the Perdew-Burke-Ernzerhof (PBE) functional within the Generalized Gradient Approximation (GGA) [44]. Van der Waals (vdW) interactions were described using the DFT-D3 method of Grimme, which incorporates a zero-damping function [45,46]. Previous studies have shown that PBE + D3 is a practical choice for investigating the adsorption behavior of transition metal dichalcogenides [47,48] and ensures the reliability of our theoretical calculations. A plane-wave cutoff energy of 500 eV was used. Monkhorst-Pack [49] k-point grids of 4 × 4 × 1 and 12 × 12 × 1 were used for structural optimization and electronic property calculations, respectively. The convergence criteria for the self-consistent field (SCF) calculations and structural optimizations were chosen as 1 × 10^−5^ eV for energy and 0.02 eV/Å for forces. The data were processed using the VASPKIT code [50]. A 4 × 4 × 1 supercell of Janus MoSTe containing 48 atoms was constructed for gas adsorption studies. A 25 Å vacuum layer was introduced along the Z-axis to prevent the effects of periodic repetitions of the structure. Furthermore, the phonon band dispersion was calculated using the small displacement method to evaluate the dynamic stability of the pristine material. Ab initio Molecular Dynamics (AIMD) simulations employed an NVT ensemble with a Nosé-Hoover thermostat to evaluate the thermodynamic stability of the pristine MoSTe. Bader charge analysis [51] was employed to investigate the charge-transfer mechanism, with the charge accumulation and depletion between gas molecules and monolayer visualized through charge density difference (CDD) plots.

The formation energy ($E_{form}$) of a surface vacancy is given by [21]:

(1) $$E_{form}=E_{defect}-E_{pristine}+\mu_{removed}$$

where $E_{defect}$ and $E_{pristine}$ denote the total energies of the monolayer with a vacancy and the pristine monolayer, respectively. $\mu_{removed}$ denotes the chemical potential of the removed atom, which is calculated by considering the bulk stable phase of the same atom.

The adsorption energy ($E_{ads}$) for a gas molecule adsorbed on the pristine (or defective) monolayer is calculated by [34]:

(2) $$E_{ads}=E_{system}-E_{monolayer}-E_{gas}$$

In the equation, $E_{system}$, $E_{monolayer}$, and $E_{gas}$ denote the total energy of the optimized adsorption system, the pristine (or defective) monolayer, and the isolated hazardous gas molecule, respectively. A negative value for $E_{ads}$ indicates a favorable exothermic adsorption process. The greater the absolute value of $E_{ads}$, the stronger the interaction between the gas molecules and the pristine (or defective) material.

The charge transfer ($Q_{trans}$) obtained from Bader charge analysis is defined by the following equation [23]:

(3) $$Q_{trans}=Q_{after-ads}-Q_{before-ads}$$

where $Q_{before-ads}$ and $Q_{after-ads}$ denote the charge of the gas molecule before and after adsorption, respectively. A positive value of $Q_{trans}$ indicates that the adsorbed gas molecule gains electrons from the pristine (or defective) monolayer, suggesting that the molecule is an electron acceptor. Conversely, a negative value of $Q_{trans}$ indicates that the gas molecule donates electrons to the pristine (or defective) monolayer, suggesting that the molecule is an electron donor.

The work function (Φ) is the minimum energy required to excite an electron from the system. The following formula is used for the calculation [20]:

(4) $$\Phi =E_{vacuum}-E_{F}$$

In the equation, $E_{vacuum}$ denotes the vacuum electrostatic potential, and $E_{F}$ denotes the Fermi level. In the electrochemical conduction of two-dimensional gas-sensitive materials, work function modulation plays a crucial role [52], and leveraging it enables the development of highly sensitive gas sensors.

The recovery time (τ) is the time required for the nanomaterial to return to its original state after adsorbing the target gas, and is calculated following the equation [53]:

(5) $$\tau =\omega^{-1}exp(\frac{{-E}_{ads}}{KT})$$

where ω is the attempt frequency, and $E_{ads}$ is the adsorption energy. *K* and *T* represent the Boltzmann constant (8.62 × 10^−5^ eV/K) and the temperature, respectively.

## 3. Results and Discussion

### 3.1. Structural and Electronic Properties of Pristine and Vacancy-Defective Janus MoSTe Monolayer

**Figure 1:**
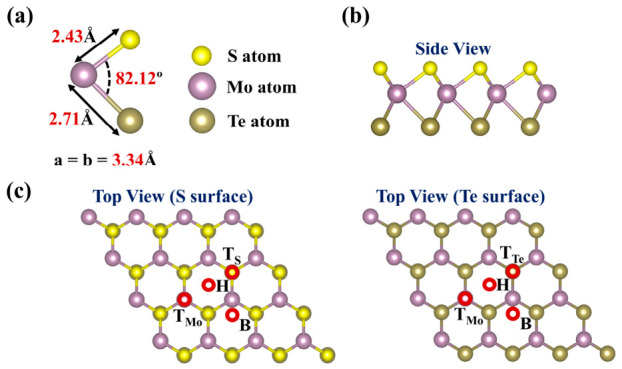
(**a**) Side view of the MoSTe unit cell, (**b**) side view of the MoSTe supercell, and (**c**) top views of the MoSTe supercell (S surface and Te surface).

**Figure 2:**
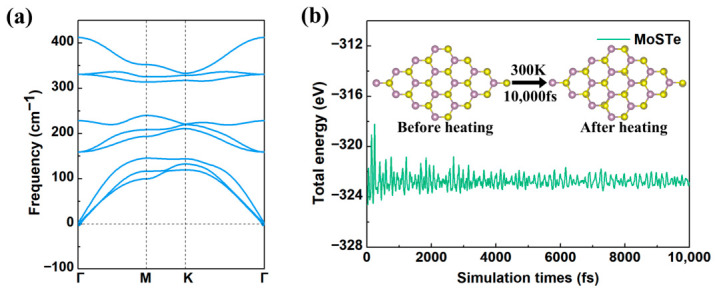
(**a**) Phonon band dispersion, and (**b**) total energy of the pristine Janus MoSTe monolayer as a function of simulation time at 300 K. The insets in (**b**) display the pristine MoSTe structures before and after heating for 10,000 fs.

The geometric structure and stability of the pristine MoSTe monolayer are first investigated. As shown in Figure 1a, the calculated lattice constant (a = b = 3.34 Å), Mo-S bond length (2.43 Å), Mo-Te bond length (2.71 Å), and the S-Mo-Te bond angle (82.12°) are consistent with previously reported findings [54,55]. The 4 × 4 × 1 supercell of pristine MoSTe contains 48 atoms (16 S, 16 Mo, and 16 Te), with a Mo layer sandwiched between S and Te layers, as observed from Figure 1b. The top views of the Janus MoSTe monolayer (Figure 1c) display a hexagonal honeycomb lattice structure. Nine vibrational modes were noted in the phonon band dispersion (Figure 2a), including three acoustic and six optical modes. The minor negative frequencies observed in the ZA (out-of-plane acoustic) mode near the Γ point are attributed to computational artifacts, resulting from slight inaccuracies in the Fast Fourier Transform (FFT) grid [56]. Previous studies have reported that the pristine Janus MoSTe exhibits a stable structure at 300 K [55,57]. To further confirm structural stability, Figure 2b shows the change in total energy versus time for the pristine Janus MoSTe monolayer at 300 K. The result shows that the structure remained intact with no bond breakage after 10,000 fs of simulation time. Therefore, the pristine Janus MoSTe monolayer demonstrates both dynamic and thermal stability, confirming its reliability as a material for studying gas molecule adsorption.

**Figure 3:**
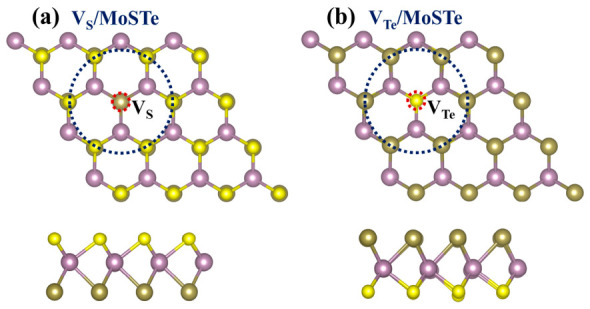
Optimized structures of the MoSTe monolayer with (**a**) an S vacancy and (**b**) a Te vacancy.

This study investigates the effect of the most common chalcogen vacancy in TMDs on gas molecule adsorption [20,58,59]. The optimized vacancy-defective structures (Figure 3) retain structural integrity. The introduction of a vacancy leads to structural reconstruction, with bond lengths around the vacancy slightly shortened (inside the blue dotted circle in Figure 3). For simplicity, the Janus MoSTe monolayer with an S vacancy is designated as V_S_/MoSTe, and the Janus MoSTe monolayer with a Te vacancy is designated as V_Te_/MoSTe. The calculated $E_{form}$ for an S vacancy is 3.17 eV, whereas that for a Te vacancy is 1.40 eV. Thus, a Te vacancy has a lower $E_{form}$ than an S vacancy, making it more readily formed. Similarly, Dou et al. observed the same phenomenon in their study of the Janus WSTe monolayer, where a Te vacancy formed more readily than an S vacancy [34].

**Figure 4:**
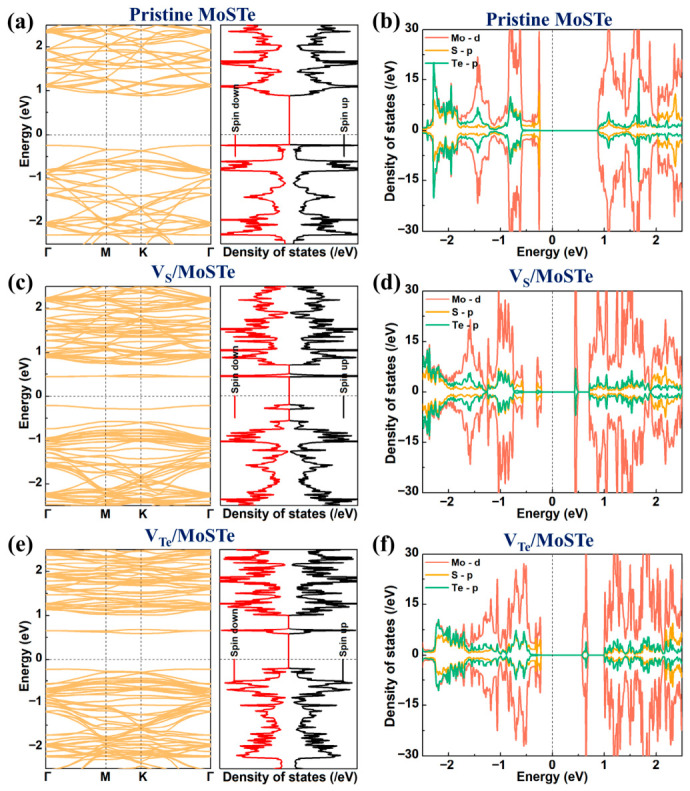
Band structures, TDOS, and PDOS for pristine and vacancy-defective Janus MoSTe monolayer: (**a**,**b**) Pristine MoSTe, (**c**,**d**) V_S_/MoSTe, (**e**,**f**) V_Te_/MoSTe. The Fermi level is set to zero.

To investigate the effect of vacancy defects on the electronic properties of MoSTe, we calculated the electronic structures of MoSTe before and after introducing a vacancy. As shown in Figure 4a, the valence band maximum (VBM) of the pristine MoSTe monolayer band structure is positioned at the Γ point, whereas the conduction band minimum (CBM) is positioned at the K point. The pristine MoSTe monolayer exhibits indirect bandgap semiconductor behavior with a band gap of 1.13 eV. Our result is close to the band gap of 1.09 eV reported by Bounbaâ et al. [60]. The total density of states (TDOS) shows symmetry between spin-up and spin-down states, indicating that the pristine MoSTe monolayer behaves as a nonmagnetic semiconductor. From the projected density of states (PDOS) (Figure 4b), it is evident that the VBM is primarily contributed by Mo-d and S-p orbitals, while the CBM is contributed by Mo-d, S-p, and Te-p orbitals. The band structures of V_S_/MoSTe and V_Te_/MoSTe (Figure 4c,e) indicate that they remain indirect bandgap semiconductors. The introduction of a vacancy defect creates defect states, thereby narrowing the band gap. The band gaps of V_S_/MoSTe and V_Te_/MoSTe decrease to 0.65 eV and 0.81 eV, respectively, thereby improving the conductivity of MoSTe. Both the spin-up and spin-down states in the TDOS of V_S_/MoSTe and V_Te_/MoSTe exhibit symmetry, supporting that they are nonmagnetic semiconductors. The PDOS of V_S_/MoSTe and V_Te_/MoSTe (Figure 4d,f) show that the defect states are mainly dominated by Mo-d, S-p, and Te-p orbitals.

### 3.2. Adsorption of Four Hazardous Gas Molecules on Pristine MoSTe Monolayer

**Figure 5:**
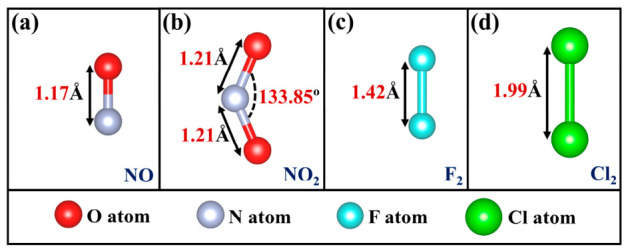
Optimized structures of hazardous gas molecules: (**a**) NO, (**b**) NO_2_, (**c**) F_2_, and (**d**) Cl_2_.

**Figure 6:**
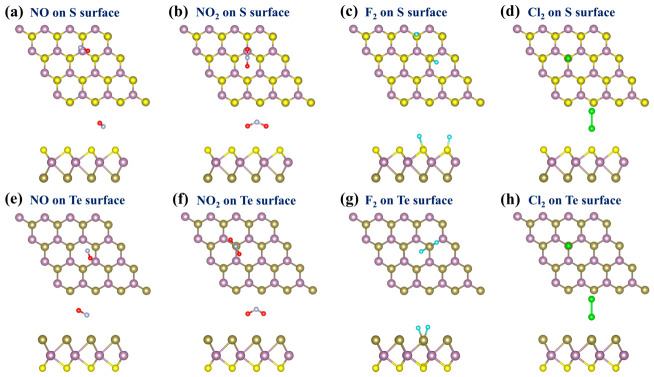
The most stable adsorption configurations for hazardous gas molecules adsorbed on the pristine MoSTe monolayer: (**a**,**e**) NO on the S/Te surface, (**b**,**f**) NO_2_ on the S/Te surface, (**c**,**g**) F_2_ on the S/Te surface, (**d**,**h**) Cl_2_ on the S/Te surface.

Unlike conventional TMDs, the structure of the Janus MoSTe monolayer is asymmetrical, where the S and Te surfaces may exhibit different effects on the adsorption of hazardous gas molecules. Therefore, two adsorption surfaces (S surface and Te surface) should be considered. As indicated in Figure 1c, this study considers four adsorption sites: (1) above the hexagonal hollow site (H site), (2) above the S-S/Te-Te bridge sites (B site), (3) above the S/Te atom (T_S_/T_Te_ site), and (4) above the Mo atom (T_Mo_ site). The relaxed structures of four hazardous gas molecules before adsorption are displayed in Figure 5, with bond lengths and bond angles consistent with previously reported values [4,61]. To ensure a comprehensive search for the most stable configuration, various typical orientations of the gas molecules were considered. For NO, three orientations were considered: (1) N atom below, O atom above (vertical orientation), (2) N atom above, O atom below (vertical orientation), and (3) NO molecule parallel to the MoSTe monolayer (parallel orientation). For NO_2_, three orientations were considered: (1) O atom as reference plane, O atom and N atom above, (2) N atom as reference plane, two O atoms above, and (3) two O atoms as reference plane, with N atom above. For F_2_ and Cl_2_, two orientations were considered: (1) F/Cl atom in a vertical orientation, and (2) F/Cl atom in a parallel orientation. Before optimizing the adsorption system, all gas molecules were initially positioned at 2.50 Å from the surface. After optimization, the most stable configurations were identified based on the minimum adsorption energy, as illustrated in Figure 6. These configurations were used for subsequent discussions of adsorption and electronic properties.

The adsorption distances of NO, NO_2_, F_2_, and Cl_2_ on the S and Te surfaces of pristine MoSTe are listed in Table 1. Except for F_2_, the adsorption distances of the other gas molecules on the S and Te surfaces are significantly greater than the sum of the single-bond covalent radii of the corresponding atoms in those gas molecules relative to the S/Te atom [62]. This indicates that NO, NO_2_, and Cl_2_ do not form chemical bonds with the pristine MoSTe monolayer. The adsorption energy of F_2_ is extremely low, at −2.07 eV and −3.31 eV on the S and Te surfaces, respectively, which is lower than the adsorption energy of −2.02 eV previously observed on pristine graphene [63]. Notably, F_2_ undergoes structural dissociation during adsorption due to strong interactions, resulting in a significantly larger bond length change (S surface: 1.42 Å to 4.50 Å, Te surface: 1.42 Å to 2.35 Å). In contrast, the adsorption energies of NO, NO_2_, and Cl_2_ on the S and Te surfaces are relatively high, spanning from −0.41 eV to −0.18 eV. Based on the adsorption distances and energies, it is tentatively concluded that F_2_ shows chemical adsorption on both surfaces of the pristine MoSTe monolayer, while NO, NO_2_, and Cl_2_ exhibit physical adsorption on both surfaces.

**Figure 7:**
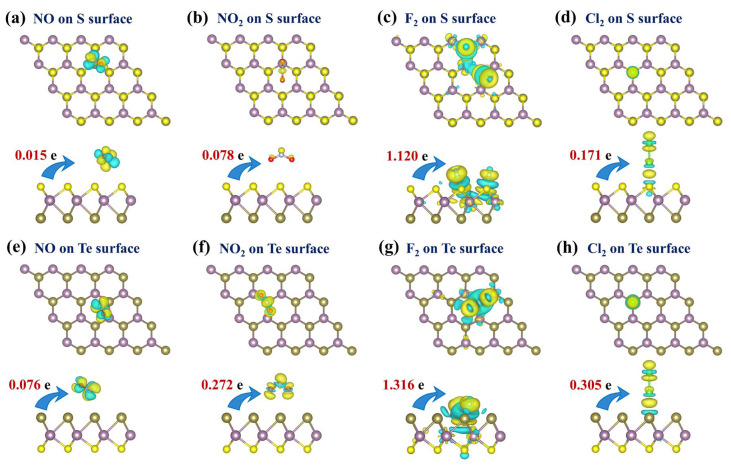
The CDD for hazardous gas molecules adsorbed on the pristine MoSTe monolayer: (**a**,**e**) NO on the S/Te surface, (**b**,**f**) NO_2_ on the S/Te surface, (**c**,**g**) F_2_ on the S/Te surface, (**d**,**h**) Cl_2_ on the S/Te surface. The isosurface value is set to 0.002 eÅ^−3^. Electron accumulation is depicted by yellow regions, and electron depletion is depicted by blue regions.

The charge density difference (CDD) of four hazardous gas molecules adsorbed on the pristine MoSTe monolayer is shown in Figure 7 and reveals that the four hazardous gas molecules serve as electron acceptors. In Table 1, Bader charge analysis offers a more precise representation of the charge transfer. It is clear that the charge transfer of F_2_ is greater than that of the other three gas molecules on both surfaces. These results arise from the strong interaction between F_2_ and the pristine MoSTe. In contrast, this also indicates that the pristine MoSTe monolayer shows weak interactions with NO, NO_2_, and Cl_2_, suggesting that it is not highly sensitive to these gases. This further demonstrates that there is a certain correlation between adsorption energy and charge transfer.

**Figure 8:**
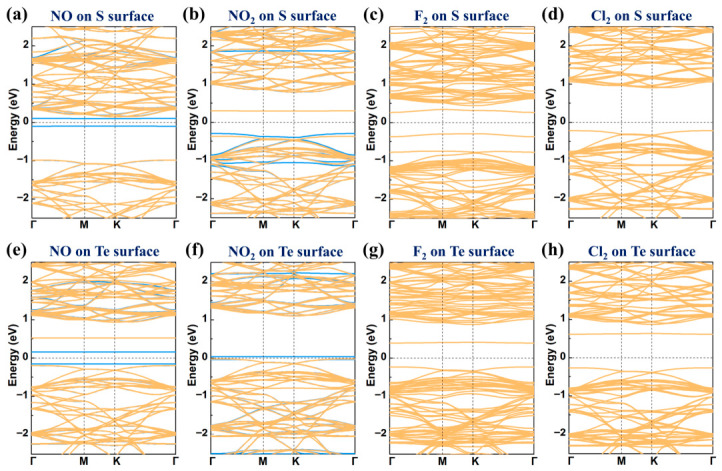
The band structures for hazardous gas molecules adsorbed on the pristine MoSTe monolayer: (**a**,**e**) NO on the S/Te surface, (**b**,**f**) NO_2_ on the S/Te surface, (**c**,**g**) F_2_ on the S/Te surface, (**d**,**h**) Cl_2_ on the S/Te surface. The blue lines represent spin-up states, and the orange lines represent spin-down states. The Fermi level is set to zero.

To further investigate the impact of gas molecule adsorption on the electronic properties of pristine MoSTe, the band structures of the adsorption of hazardous gas molecules on the pristine MoSTe monolayer were calculated, as shown in Figure 8. The corresponding band gaps of these systems are provided in Table 1. For the adsorption of NO and NO_2_ on the pristine MoSTe monolayer, the spin-up and spin-down states in the band structure do not overlap, indicating that the adsorption of NO and NO_2_ introduces magnetism into the system. This is consistent with the previously observed adsorption of NO and NO_2_ on materials such as g-GaN [64], WS_2_ [22], and MoSi_2_N_4_ [65]. Additionally, significant changes near the Fermi level lead to a substantial narrowing of the bandgap relative to the pristine MoSTe (1.13 eV). When NO and NO_2_ adsorb on the S surface, their band gaps decrease to 0.20 eV and 0.59 eV, respectively. On the Te surface, the band gaps decrease to 0.31 eV and 0.05 eV, respectively. The four systems are indirect bandgap semiconductors. In the case of F_2_ and Cl_2_ adsorption, the adsorption systems remain nonmagnetic. When F_2_ and Cl_2_ adsorb on the S surface, the band gaps of the adsorbed systems are 0.56 eV and 1.12 eV, respectively, and the adsorbed systems retain indirect bandgap semiconductors. However, when adsorbed on the Te surface, the band gaps change to 0.62 eV and 0.88 eV, respectively. Excitingly, the F_2_ and Cl_2_ adsorbed systems on the Te surface transform into direct bandgap semiconductors.

**Figure 9:**
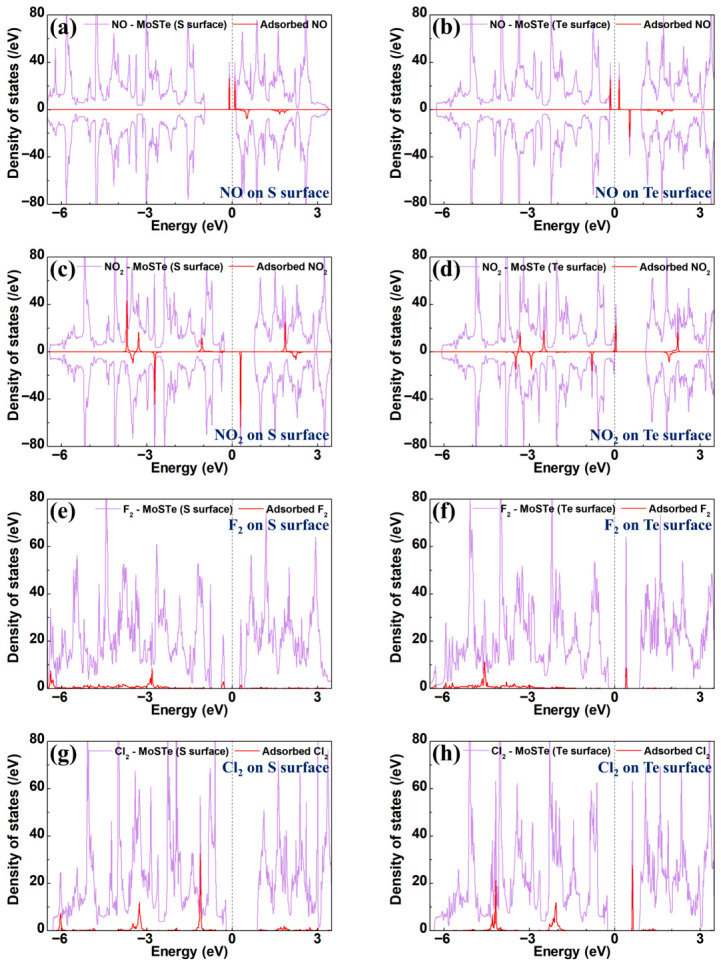
The TDOS for hazardous gas molecules adsorbed on the pristine MoSTe monolayer: (**a**,**b**) NO on the S/Te surface, (**c**,**d**) NO_2_ on the S/Te surface, (**e**,**f**) F_2_ on the S/Te surface, (**g**,**h**) Cl_2_ on the S/Te surface. The Fermi level is set to zero.

The TDOS for four hazardous gas molecules adsorbed on the pristine MoSTe monolayer is shown in Figure 9, with the corresponding PDOS provided in Figure S3. For NO adsorption on S and Te surfaces, the sharp peaks close to the Fermi level originate from impurity states induced by NO adsorption, which correspond to the appearance of two flat bands close to the Fermi level in the band structure. The PDOS indicates that the introduction of magnetism is primarily resulting from the N-p and O-p orbitals. Furthermore, no orbital hybridization occurs near the Fermi level. This phenomenon, combined with the relatively high adsorption energy and small charge transfer, further confirms the physisorption of NO on the pristine MoSTe. For NO_2_ adsorption, when NO_2_ adsorbs on the S surface, impurity states appear in the conduction band (CB). Notably, when NO_2_ adsorbs on the Te surface, it exhibits an extremely narrow band gap due to impurity states close to the Fermi level. The PDOS shows no evidence of orbital hybridization, indicating no strong interaction between the NO_2_ gas molecule and the material. This is consistent with the physisorption exhibited by the adsorption distance and energy. Similar to NO adsorption, its magnetism primarily originates from the influence of the N-p and O-p orbitals. Regarding the F_2_ adsorbed on both surfaces, the contribution of the F_2_ electronic states to the TDOS is primarily within the range of −6.5 eV to −2.5 eV, and introduces impurity states near the Fermi level. The PDOS reveals significant orbital overlap between the F-p orbital and the S-p/Te-p orbitals, indicating strong interaction between the F_2_ molecule and the MoSTe monolayer, thereby confirming the chemisorption of F_2_ on the pristine MoSTe. For Cl_2_ adsorbed on the S surface, no contribution from Cl_2_ electronic states is observed between the VBM and CBM, leading to negligible changes to the band gap. However, on the Te surface, a peak in the CB at 0.64 eV significantly influences the band gap. The PDOS analysis indicates that no orbital hybridization occurs. However, the relatively low adsorption energy and relatively big charge transfer suggest that the adsorption of Cl_2_ on the pristine MoSTe is a strong physisorption.

### 3.3. Adsorption of Four Hazardous Gas Molecules on Vacancy-Defective MoSTe Monolayer

**Figure 10:**
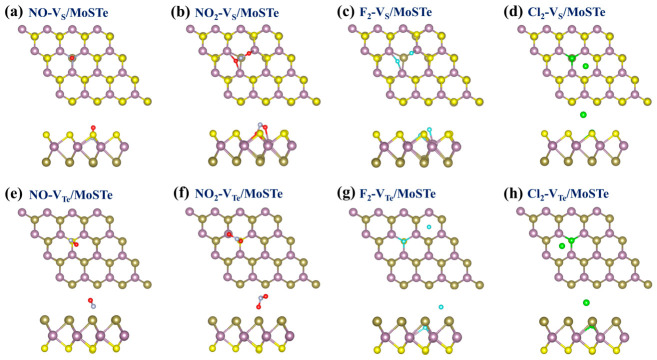
The most stable adsorption configurations for hazardous gas molecules adsorbed on vacancy-defective MoSTe monolayer: (**a**) NO-V_S_/MoSTe, (**b**) NO_2_-V_S_/MoSTe, (**c**) F_2_-V_S_/MoSTe, (**d**) Cl_2_-V_S_/MoSTe, (**e**) NO-V_Te_/MoSTe, (**f**) NO_2_-V_Te_/MoSTe, (**g**) F_2_-V_Te_/MoSTe, (**h**) Cl_2_-V_Te_/MoSTe.

Since our primary focus is on the impact of vacancy defects on hazardous gas molecule adsorption, we consider only the adsorption site directly above the vacancy defect (as highlighted by the red dashed circle in Figure 3). The orientations considered for the four hazardous gas molecules are consistent with those considered for molecules adsorbed on the pristine MoSTe monolayer, and the initial adsorption distance remains 2.50 Å. The configuration with the minimum adsorption energy for each gas molecule was selected for subsequent studies, as depicted in Figure 10. It can be observed that for NO adsorbed on both V_S_/MoSTe and V_Te_/MoSTe, the N atom is close to the adsorption surface, while the O atom moves away from it. For NO_2_ adsorption on V_S_/MoSTe and V_Te_/MoSTe, the configurations differ from those of NO: The O atom tends to approach the adsorption surface, while the N atom tends to move away from it. Regarding F_2_ and Cl_2_ on V_S_/MoSTe and V_Te_/MoSTe, it is noteworthy that these two gas molecules dissociate upon adsorption, splitting into two halogen atoms. This is consistent with previous findings showing that F_2_ and Cl_2_ dissociate into two atoms when adsorbed onto borophene [66].

The adsorption distances and energies of each hazardous gas molecule on vacancy-defective MoSTe are presented in Table 2. For the adsorption of NO and NO_2_ on the V_S_/MoSTe, the adsorption distances are significantly shorter than those on the S surface of the pristine MoSTe, and the adsorption energies are significantly lower. In contrast, there is little change in the adsorption distances and adsorption energies on the V_Te_/MoSTe compared to adsorption on the Te surface of pristine MoSTe. Based on adsorption distances and adsorption energies, this suggests that NO and NO_2_ exhibit distinct adsorption behaviors on the two vacancy types: on V_S_/MoSTe, they undergo chemical adsorption, whereas on V_Te_/MoSTe, they undergo strong physical adsorption. The adsorption energies of F_2_ and Cl_2_ on vacancy-defective Janus MoSTe are significantly lower than those on pristine MoSTe. Additionally, the analysis of adsorption distances and adsorption energies reveals that F_2_ and Cl_2_ exhibit strong chemical adsorption on both vacancy types.

**Figure 11:**
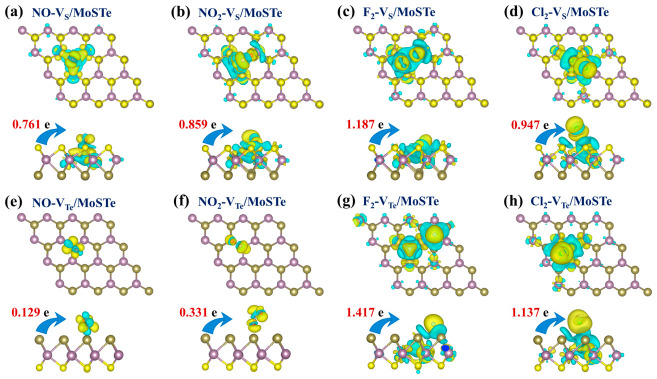
The CDD for hazardous gas molecules adsorbed on the vacancy-defective MoSTe monolayer: (**a**) NO-V_S_/MoSTe, (**b**) NO_2_-V_S_/MoSTe, (**c**) F_2_-V_S_/MoSTe, (**d**) Cl_2_-V_S_/MoSTe, (**e**) NO-V_Te_/MoSTe, (**f**) NO_2_-V_Te_/MoSTe, (**g**) F_2_-V_Te_/MoSTe, (**h**) Cl_2_-V_Te_/MoSTe. The isosurface value is set to 0.002 eÅ^−3^. Electron accumulation is depicted by yellow regions, and electron depletion is depicted by blue regions.

The CDD for the adsorption of four hazardous gas molecules on the V_S_/MoSTe and V_Te_/MoSTe are presented in Figure 11. On the V_S_/MoSTe, NO, NO_2_, F_2_, and Cl_2_ transfer charges of 0.761 e, 0.859 e, 1.187 e, and 0.947 e from the material, respectively. This indicates that these hazardous gas molecules exhibit similar acceptor properties to those adsorbed on pristine MoSTe. After the introduction of an S vacancy, charge transfer for NO, NO_2_, F_2_, and Cl_2_ all increase to varying degrees. On the V_Te_/MoSTe, the charge transfer for NO, NO_2_, F_2_, and Cl_2_ is 0.129 e, 0.331 e, 1.417 e, and 1.137 e, respectively, all of which maintain their acceptor properties. Compared to adsorption on the Te surface of the pristine MoSTe monolayer, the charge transfer for NO, NO_2_, and F_2_ increases slightly on the V_Te_/MoSTe, whereas that for Cl_2_ increases significantly. Overall, the introduction of vacancy enhances charge transfer, which may improve the sensitivity of gas sensing and detection.

**Figure 12:**
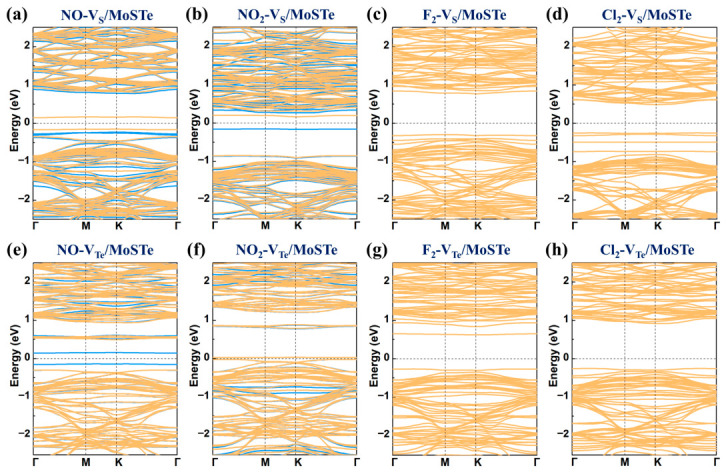
The band structures for hazardous gas molecules adsorbed on the vacancy-defective MoSTe monolayer: (**a**) NO-V_S_/MoSTe, (**b**) NO_2_-V_S_/MoSTe, (**c**) F_2_-V_S_/MoSTe, (**d**) Cl_2_-V_S_/MoSTe, (**e**) NO-V_Te_/MoSTe, (**f**) NO_2_-V_Te_/MoSTe, (**g**) F_2_-V_Te_/MoSTe, (**h**) Cl_2_-V_Te_/MoSTe. The blue lines represent spin-up states, and the orange lines represent spin-down states. The Fermi level is set to zero.

The band structures for the adsorption of four hazardous gas molecules on V_S_/MoSTe and V_Te_/MoSTe are demonstrated in Figure 12. For NO and NO_2_ adsorbed on V_S_/MoSTe and V_Te_/MoSTe, the spin-up and spin-down states in the band structures exhibit non-overlapping. This indicates these systems are magnetic semiconductors, similar to those observed for adsorption on the pristine MoSTe monolayer. Notably, in contrast to the adsorption of NO on the S surface of pristine MoSTe monolayer, where the VBM and CBM are primarily contributed by the spin-up state, the spin-down state dominates in the VBM and CBM of NO-V_S_/MoSTe. For the NO-V_Te_/MoSTe and NO adsorbed on the Te surface of the pristine MoSTe monolayer, both the VBM and CBM are primarily contributed by the spin-up state. As shown in Table 2, the band gaps for NO-V_S_/MoSTe, NO-V_Te_/MoSTe, NO_2_-V_S_/MoSTe, and NO_2_-V_Te_/MoSTe are 0.30 eV, 0.28 eV, 0.31 eV, and 0.05 eV, respectively. These values are significantly smaller than the band gaps of V_S_/MoSTe (0.65 eV) and V_Te_/MoSTe (0.81 eV), indicating that gas adsorption can modulate the band gaps. NO-V_S_/MoSTe, NO-V_Te_/MoSTe, NO_2_-V_S_/MoSTe, and NO_2_-V_Te_/MoSTe are all indirect semiconductors. This behavior is consistent with that observed on the pristine MoSTe monolayer. Regarding the adsorption of F_2_ and Cl_2_, the systems adsorbed on both V_S_/MoSTe and V_Te_/MoSTe remain indirect semiconductors. The band gaps of F_2_-V_S_/MoSTe, F_2_-V_Te_/MoSTe, Cl_2_-V_S_/MoSTe, and Cl_2_-V_Te_/MoSTe are all larger than the band gaps of the unadsorbed V_S_/MoSTe and V_Te_/MoSTe, which may be unfavorable for conductivity. Therefore, the adsorption of these hazardous gas molecules can modulate the electronic properties of the vacancy-defective MoSTe monolayer.

**Figure 13:**
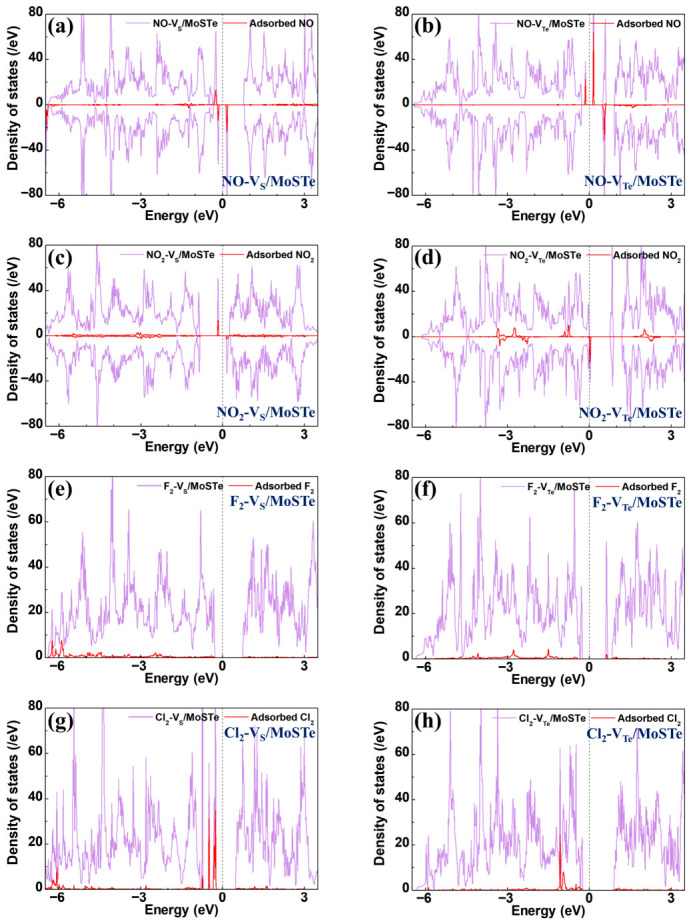
The TDOS for hazardous gas molecules adsorbed on the vacancy-defective MoSTe monolayer: (**a**) NO-V_S_/MoSTe, (**b**) NO-V_Te_/MoSTe, (**c**) NO_2_-V_S_/MoSTe, (**d**) NO_2_-V_Te_/MoSTe, (**e**) F_2_-V_S_/MoSTe, (**f**) F_2_-V_Te_/MoSTe, (**g**) Cl_2_-V_S_/MoSTe, (**h**) Cl_2_-V_Te_/MoSTe. The Fermi level is set to zero.

The TDOS of four hazardous gas molecules adsorbed on V_S_/MoSTe and V_Te_/MoSTe are presented in Figure 13, with the corresponding PDOS provided in Figure S4. The TDOS for the NO-V_S_/MoSTe, NO-V_Te_/MoSTe, NO_2_-V_S_/MoSTe, and NO_2_-V_Te_/MoSTe exhibit spin-up and spin-down asymmetry, indicating that these systems are magnetic semiconductors. These results align with the band structure analysis. For NO adsorption, the NO electronic states show distinct peaks on either side of the Fermi level, which directly leads to a reduction in the band gap. For NO_2_-V_S_/MoSTe, the electronic states of adsorbed NO_2_ show peaks at −0.15 eV and 0.17 eV. In contrast, for the NO_2_-V_Te_/MoSTe system, the electronic states of NO_2_ primarily contribute between −3.5 eV and −2.0 eV, and near the Fermi level. Notably, significant orbital hybridization is observed in the PDOS of NO-V_S_/MoSTe and NO_2_-V_S_/MoSTe, while no orbital hybridization is observed for NO-V_Te_/MoSTe and NO_2_-V_Te_/MoSTe. This further confirms that NO and NO_2_ undergo chemisorption on V_S_/MoSTe, while undergoing physisorption on V_Te_/MoSTe. For the F_2_-V_S_/MoSTe system, the defect states in the CB vanish due to the dissociation of the F_2_ at the defect sites. The PDOS indicates orbital overlap between the gas molecular states and the substrate states. Similar orbital overlap is observed in the F_2_-V_Te_/MoSTe system, further confirming that F_2_ undergoes chemisorption on the vacancy-defective Janus MoSTe. For the Cl_2_ adsorption system, the electronic states of Cl_2_ primarily contribute within the VB. The PDOS analysis indicates significant orbital hybridization near the Fermi level, which has a significant impact on the electronic properties of the defective Janus MoSTe. This confirms that Cl_2_ undergoes chemisorption on the vacancy-defective Janus MoSTe.

### 3.4. Work Function for the Adsorption of Four Hazardous Gas Molecules on Vacancy-Defective MoSTe Monolayer

**Figure 14:**
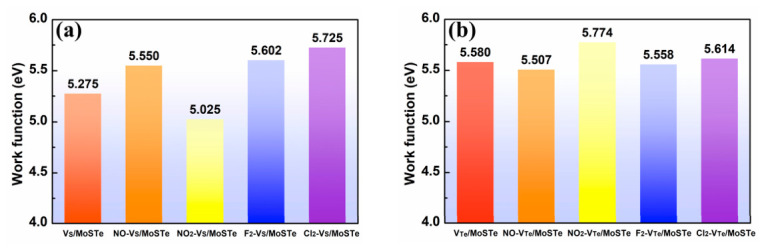
The work function of four hazardous gas molecules on vacancy-defective MoSTe monolayer before and after adsorption: (**a**) V_S_/MoSTe and (**b**) V_Te_/MoSTe.

The work functions (Φ) of vacancy-defective MoSTe monolayer before and after adsorption are shown in Figure 14. The calculated work function of the pristine MoSTe is 5.627 eV (Figure S2), while for V_S_/MoSTe and V_Te_/MoSTe, the work functions are 5.275 eV and 5.580 eV, respectively. The work function decreases following the removal of a negatively charged S and Te atom. This phenomenon was also observed in previous studies on HfSeTe, where the removal of negatively charged Se or Te atoms lowered the work function [21]. The work function undergoes a change following the adsorption of hazardous gas molecules on V_S_/MoSTe and V_Te_/MoSTe. The adsorption of NO, F_2_, and Cl_2_ on V_S_/MoSTe increased the work function to 5.550 eV, 5.602 eV, and 5.725 eV, respectively, whereas NO_2_ adsorption decreased it to 5.025 eV, indicating that gas adsorption induced different work function responses. On V_Te_/MoSTe, the adsorption of NO and F_2_ decreases the work function, whereas NO_2_ and Cl_2_ increase it. This indicates that, following the adsorption of NO and F_2_, electron desorption becomes easier, whereas following the adsorption of NO_2_ and Cl_2_, electron transfer becomes difficult, further illustrating that different gas molecules impact charge-transfer behavior. The above results indicate that vacancy-defective Janus MoSTe exhibits differentiated work function responses to different gas molecules, reflecting the differences in interactions between the gas molecules and the material surface. The results of DFT calculations, which include adsorption energy, charge transfer, and work function responses induced by gas adsorption, suggest that vacancy-defective Janus MoSTe may exhibit a certain degree of sensitivity for hazardous gases. However, these DFT models make assumptions about specific defect densities and gas coverage that may not accurately reflect actual experimental conditions. Therefore, the conclusion should be regarded merely as a qualitative insight rather than a definitive prediction of actual performance.

### 3.5. Recovery Time for the Adsorption of Four Hazardous Gas Molecules on Vacancy-Defective MoSTe Monolayer

Table 3 shows the recovery times (τ) of four hazardous gas molecules adsorbed on vacancy-defective Janus MoSTe with exposure to visible light (ω = 10^13^ s^−1^) [53] at different temperatures (300 K, 400 K, and 500 K), and the recovery times (τ) of four hazardous gas molecules adsorbed on the V_S_/MoSTe and of F_2_ and Cl_2_ adsorbed on the V_Te_/MoSTe under UV light irradiation (ω = 10^16^ s^−1^) [67] at 500K. It can be seen that NO and NO_2_ exhibit short recovery times on V_Te_/MoSTe at 300 K, at 1.61 × 10^−8^ s and 1.11 × 10^−7^ s, respectively. This indicates that these gas molecules desorb easily from the V_Te_/MoSTe. However, at room temperature, the adsorption energy of four hazardous gas molecules on V_S_/MoSTe and of F_2_ and Cl_2_ on V_Te_/MoSTe is lower, resulting in longer recovery times that exceed practical operational limits. This means that under room-temperature conditions, the adsorption process is essentially irreversible. In most cases, increasing the temperature and adjusting the attempt frequency are effective ways to reduce recovery time [53,68,69,70]. As shown in Table 3, the recovery time for all adsorption systems decreases significantly as temperature increases. In particular, for NO_2_-V_S_/MoSTe, the recovery time was reduced to 6.38 s under visible light at 500 K, and to 6.38 × 10^−3^ s under UV light at 500 K. Therefore, at the relatively high temperature of 500 K and under UV light irradiation, the recovery time of NO_2_ adsorbed on V_S_/MoSTe can be reduced to an acceptable level. For the adsorption of F_2_ and Cl_2_ on V_S_/MoSTe and V_Te_/MoSTe, and of NO on V_S_/MoSTe, heating to 500 K and UV light irradiation do indeed shorten the recovery time, but they remain unacceptable for practical applications. This indicates that these adsorption systems are difficult to recover.

### 3.6. Adsorption of O_2_ Molecule on Vacancy-Defective MoSTe Monolayer

**Figure 15:**
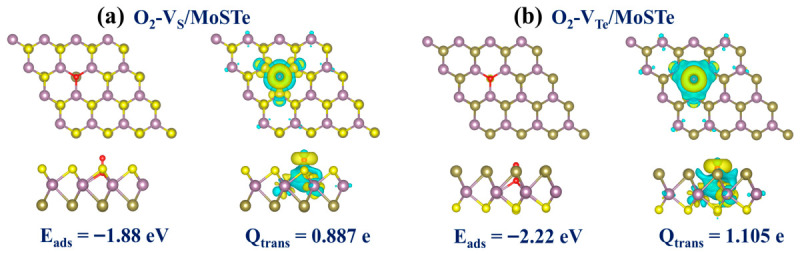
The adsorption configurations and CDD for O_2_ adsorbed on vacancy-defective MoSTe monolayer: (**a**) O_2_-V_S_/MoSTe, (**b**) O_2_-V_Te_/MoSTe. For CDD, the isosurface value is set to 0.002 eÅ^−3^. Electron accumulation is depicted by yellow regions, and electron depletion is depicted by blue regions.

Although the above results indicate that the interaction between hazardous gas molecules and vacancy-defective Janus MoSTe is enhanced compared to that between the gas molecules and the pristine Janus MoSTe, it is worth noting that the adsorption of the O_2_ molecule on the vacancy-defective MoSTe monolayer still needs to be considered. In practical gas sensing applications, most are conducted in aerobic environments. Numerous previous studies have shown that chalcogen vacancies in TMDs are highly reactive toward O_2_ and readily decompose into metal oxides or are passivated by O_2_ [71,72,73,74]. Therefore, we performed theoretical calculations on the adsorption of O_2_ at S and Te vacancies in Janus MoSTe to evaluate its stability in aerobic environments. As shown in Figure 15, the calculated adsorption energies of O_2_ at the S and Te vacancies are −1.88 eV and −2.22 eV, respectively, and the transferred charges are 0.887 e and 1.105 e, respectively. These values indicate a strong interaction between O_2_ and vacancy-defective Janus MoSTe. Based on the adsorption configuration, O_2_ tends to adopt a vertical orientation, with one O atom bonding to exposed Mo atoms while the other points outward. Therefore, the S and Te vacancies in Janus MoSTe are occupied by O_2_. This phenomenon is consistent with previous reports on MoS_2_ and MoTe_2_ [75,76]. This may alter or even suppress the adsorption behavior of vacancy-defective Janus MoSTe monolayer toward target gas molecules, posing a significant challenge for sensing applications under atmospheric conditions.

To avoid the impact of O_2_ adsorption, we assume vacancy-defective Janus MoSTe is used in oxygen-free environments. Given the acceptable recovery times of NO-V_Te_/MoSTe and NO_2_-V_Te_/MoSTe at room temperature, V_Te_/MoSTe may be used to sense NO and NO_2_. However, the recovery times for other adsorption systems at room temperature exceed practical limits. Regarding the adsorption of NO_2_ on V_S_/MoSTe, although heating and UV irradiation reduced the recovery time of this adsorption system to an acceptable level, it is important to note that these methods may have limitations in practical applications. Excessively high temperatures or irradiation intensities are likely to cause the decomposition of Janus MoSTe under real conditions. Furthermore, previous studies on MoTe_2_ have shown that surface dissociation takes place at 200 °C, leading to the formation of Te vacancies, pinholes, and randomly shaped clusters, and as the temperature rises, these defects gradually increase [77]. Since MoSTe contains Te, it is likely to exhibit this phenomenon as well, which imposes practical limitations on its heat treatment temperature range and poses challenges for its practical sensing applications. For the adsorption of F_2_ and Cl_2_ on V_S_/MoSTe and V_Te_/MoSTe, and of NO on V_S_/MoSTe, the recovery time remains unacceptable even under UV irradiation at 500 K, which poses a challenge to their reusability in sensing applications. Consequently, the issue of recovery time also limits its practical sensing potential. It is possible that, in scenarios where oxygen-free and high-temperature recovery does not affect the material’s stability, vacancy-defective Janus MoSTe may be used for hazardous gas sensing.

## 4. Conclusions

This study systematically investigates the adsorption behavior of four hazardous gas molecules (NO, NO_2_, F_2_, and Cl_2_) on pristine and vacancy-defective Janus MoSTe monolayers. F_2_ exhibits chemisorption on S and Te surfaces of the pristine MoSTe, with adsorption energies of −2.07 eV and −3.31 eV, respectively, whereas other gases undergo physisorption on the pristine MoSTe, with adsorption energies ranging from −0.41 eV to −0.18 eV. Gas adsorption reduces the band gap of pristine MoSTe, while adsorption of F_2_ and Cl_2_ on the Te surface induces an indirect-to-direct bandgap transition. Additionally, NO and NO_2_ adsorption introduce magnetic properties into the system. The introduction of an S vacancy changes the adsorption energies of NO, NO_2_, and Cl_2_ to −2.73 eV, −1.37 eV, and −2.70 eV, respectively, and increases the charge transfers to 0.761 e, 0.859 e, and 0.947 e, causing them to transition from physisorption to chemisorption, thereby enhancing the strength of gas adsorption. Notably, NO and NO_2_ adsorption at vacancy sites narrows the band gap, whereas F_2_ and Cl_2_ adsorption widens it. Work function analysis suggests that, under ideal conditions, vacancy-defective Janus MoSTe may exhibit sensitivity to hazardous gases. The desorption of four gas molecules from vacancy-defective Janus MoSTe was reasonably assessed by using the recovery time. By studying the adsorption behavior of O_2_ on vacancy-defective Janus MoSTe, we have realized the challenges posed by oxygen passivation under atmospheric conditions, which may alter or even suppress the adsorption of target gas molecules. Overall, this study provides a comprehensive analysis of the hazardous gas adsorption properties of vacancy-defective Janus MoSTe, elucidating the limitations of its sensing potential and the practical challenges it faces under real sensing conditions, and offering valuable theoretical insights and guidance for future experiments on this material.

## Figures and Tables

**Table 1:** Adsorption distance, adsorption energy, charge transfer, and band gap for NO, NO_2_, F_2_, and Cl_2_ gas molecules adsorbed on the S and Te surfaces of the pristine MoSTe monolayer.

Surface	Gas Molecule	Adsorption Distance d (Å)	Adsorption Energy E_ads_ (eV)	Charge Transfer Q_trans_ (e)	Band Gap E_g_ (eV)
S Surface	NO	3.22	−0.18	0.015	0.20
	NO_2_	3.34	−0.22	0.078	0.59
	F_2_	1.71	−2.07	1.120	0.56
	Cl_2_	2.60	−0.35	0.171	1.12
Te Surface	NO	3.16	−0.20	0.076	0.31
	NO_2_	3.31	−0.30	0.272	0.05
	F_2_	2.02	−3.31	1.316	0.62
	Cl_2_	2.77	−0.41	0.305	0.88

**Table 2:** Adsorption distance, adsorption energy, charge transfer, and band gap for NO, NO_2_, F_2_, and Cl_2_ gas molecules adsorbed on vacancy-defective MoSTe monolayer.

Model	Gas Molecule	Adsorption Distance d (Å)	Adsorption Energy E_ads_ (eV)	Charge Transfer Q_trans_ (e)	Band Gap E_g_ (eV)
V_S_/MoSTe	NO	2.18	−2.73	0.761	0.30
	NO_2_	2.21	−1.37	0.859	0.31
	F_2_	1.98	−5.63	1.187	1.06
	Cl_2_	2.51	−2.70	0.947	0.74
V_Te_/MoSTe	NO	3.41	−0.31	0.129	0.28
	NO_2_	3.57	−0.36	0.331	0.05
	F_2_	2.22	−5.54	1.417	0.89
	Cl_2_	2.42	−2.72	1.137	1.17

**Table 3:** Recovery time of four hazardous gas molecules on vacancy-defective MoSTe monolayer.

Model	Gas Molecule	Recovery Time τ (s)			
		300 K (Visible Light)	400 K (Visible Light)	500 K (Visible Light)	500 K (UV Light)
V_S_/MoSTe	NO	7.04 × 10^32^	2.43 × 10^21^	3.23 × 10^14^	3.23 × 10^11^
	NO_2_	1.02 × 10^10^	1.80 × 10^4^	6.38	6.38 × 10^−3^
	F_2_	3.55 × 10^81^	8.18 × 10^57^	5.37 × 10^43^	5.37 × 10^40^
	Cl_2_	2.21 × 10^32^	1.02 × 10^21^	1.61 × 10^14^	1.61 × 10^11^
V_Te_/MoSTe	NO	1.61 × 10^−8^	8.03 × 10^−10^	1.33 × 10^−10^	—
	NO_2_	1.11 × 10^−7^	3.42 × 10^−9^	4.24 × 10^−10^	—
	F_2_	1.09 × 10^80^	6.02 × 10^56^	6.66 × 10^42^	6.66 × 10^39^
	Cl_2_	4.78 × 10^32^	1.82 × 10^21^	2.56 × 10^14^	2.56 × 10^11^

## Data Availability

The original contributions presented in this study are included in the article/Supplementary Materials. Further inquiries can be directed to the corresponding author.

## Supplementary Materials

The following supporting information can be downloaded at: https://www.mdpi.com/article/10.3390/nano16100621/s1, Figure S1: The Bader charges of pristine (a) MoSTe (S surface and Te surface), (b) MoS_2_, and (c) MoTe_2_; Figure S2: Plane-averaged electrostatic potential of the pristine MoSTe monolayer; Figure S3: The PDOS for hazardous gas molecules adsorbed on the pristine MoSTe monolayer: (a,b) NO on the S/Te surface, (c,d) NO_2_ on the S/Te surface, (e,f) F_2_ on the S/Te surface, (g,h) Cl_2_ on the S/Te surface. The Fermi level is set to zero; Figure S4: The PDOS for hazardous gas molecules adsorbed on the vacancy-defective MoSTe monolayer: (a) NO-V_S_/MoSTe, (b) NO-V_Te_/MoSTe, (c) NO_2_-V_S_/MoSTe, (d) NO_2_-V_Te_/MoSTe, (e) F_2_-V_S_/MoSTe, (f) F_2_-V_Te_/MoSTe, (g) Cl_2_-V_S_/MoSTe, (h) Cl_2_-V_Te_/MoSTe. The Fermi level is set to zero.
