# Supplementary material for: First-Principles Study of Hazardous Gas Molecule Adsorption on Janus MoSTe Monolayer Modified with Surface Vacancy Defect
Source: Nanomaterials (Basel). 2026 May 18;16(10):621. doi: 10.3390/nano16100621 (PMC13209916; doi:10.3390/nano16100621)
Supplement: Supplementary file 1 [file nanomaterials-16-00621-s001.zip › nanomaterials-4263565-supplementary.pdf]

First-Principles Study of Hazardous Gas Molecule Adsorption on Janus MoSTe Monolayer Modified with Surface Vacancy Defect

Supplementary Materials

Yuhui Zhu [1], Sheng Xu [1,*], Qiang Wang [1], Yanni Gu [1], Xiaoli Zhang [1] and Xiaoshan Wu [2]

[1] School of Metallurgy Engineering, Jiangsu University of Science and Technology, Zhangjiagang 215600, China; E-mail: xsl258@just.edu.cn

[2] National Laboratory of Solid State Microstructures and School of Physics, Nanjing University, Nanjing 210093, China

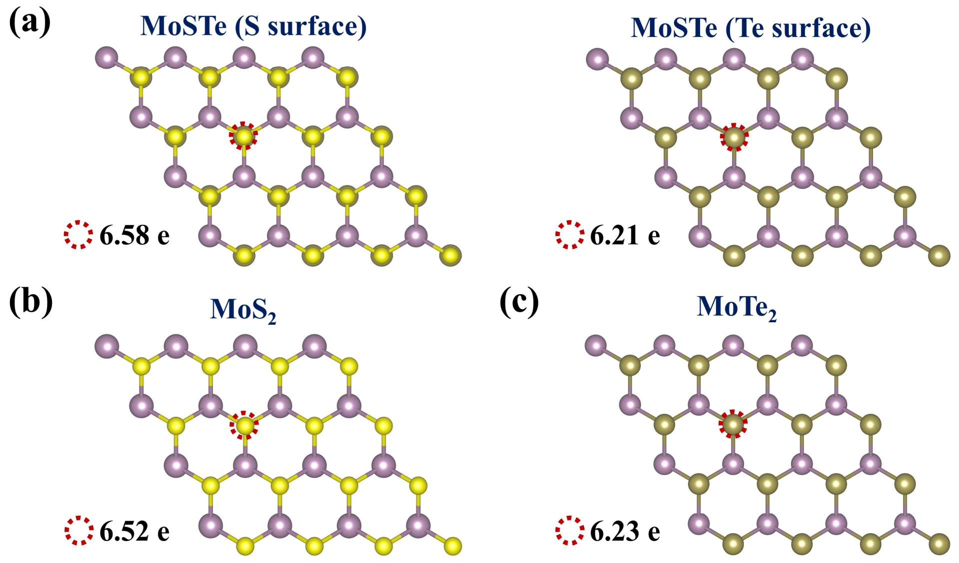

Figure S1. The Bader charges of pristine (**a**) MoSTe (S surface and Te surface), (**b**) MoS_2, and (**c**) $MoTe_2$.

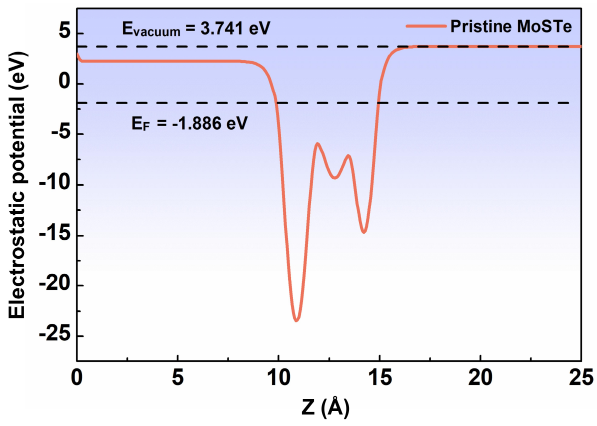

Figure S2. Plane-averaged electrostatic potential of the pristine MoSTe monolayer.

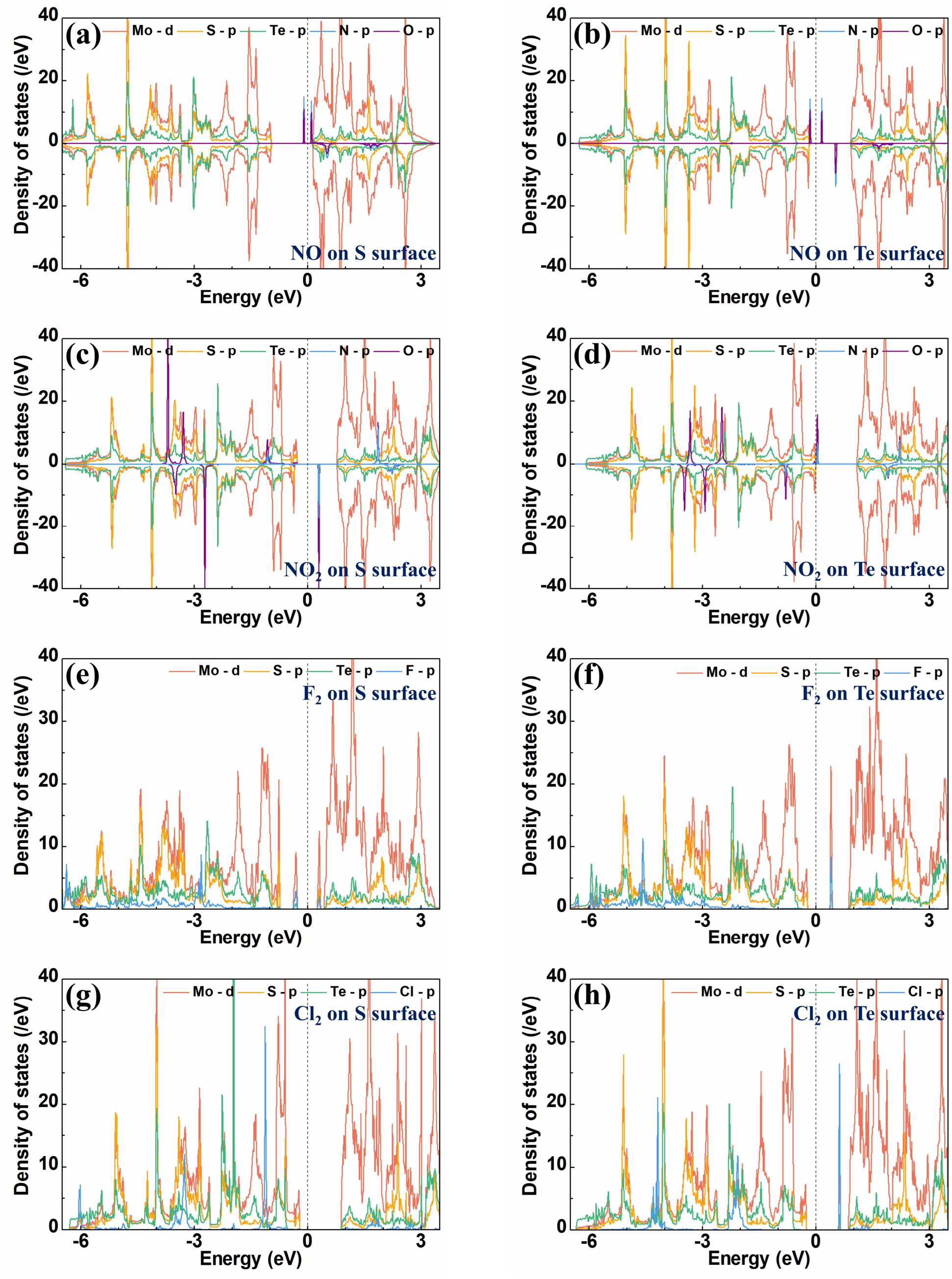

Figure S3. The PDOS for hazardous gas molecules adsorbed on the pristine MoSTe monolayer: (**a**,**b**) NO on the S/Te surface, (**c**,**d**) NO_2 on the S/Te surface, (**e**,**f**) F_2 on the S/Te surface, (**g**,**h**) Cl_2 on the S/Te surface. The Fermi level is set to zero.

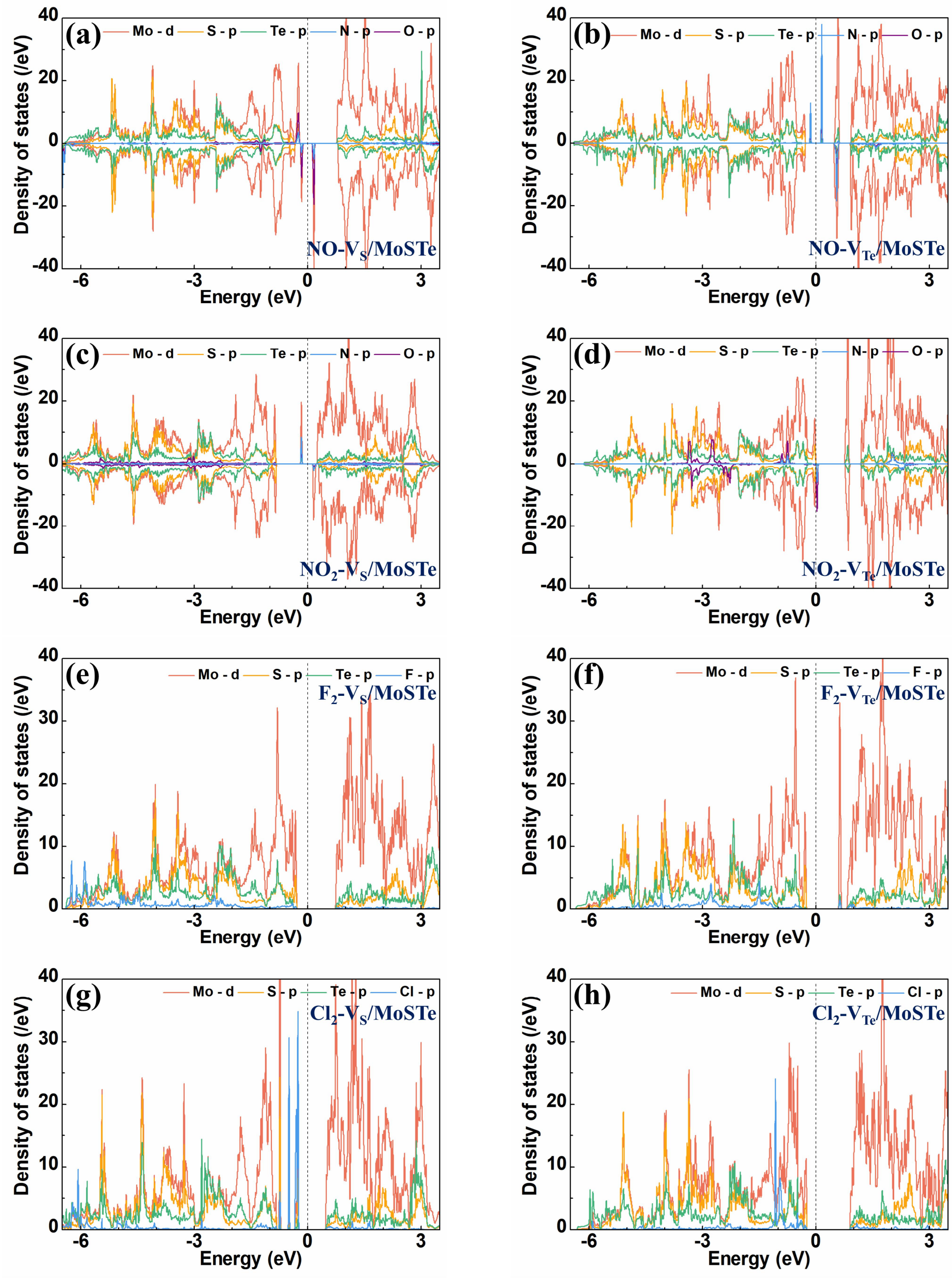

Figure S4. The PDOS for hazardous gas molecules adsorbed on the vacancy-defective MoSTe monolayer: (**a**) NO-V_S/MoSTe, (**b**) NO-V_{Te}/MoSTe, (**c**) NO_2-V_S/MoSTe, (**d**) NO_2-V_{Te}/MoSTe, (**e**) F_2-V_S/MoSTe, (**f**) F_2-V_{Te}/MoSTe, (**g**) Cl_2-V_S/MoSTe, (**h**) Cl_2-V_{Te}/MoSTe. The Fermi level is set to zero.
